# Case Report: Cytomegalovirus retinitis in an HIV-negative patient with splenic mantle cell lymphoma treated with bendamustine-rituximab

**DOI:** 10.3389/fimmu.2026.1979881

**Published:** 2026-10-28

**Authors:** Yi-Hsien Tsai, Hsien-Chung Lin, Wen-Chi Yang, Ta-Jen Lin, Jih-Jin Tsai

**Affiliations:** 1 School of Post-Baccalaureate Medicine, College of Medicine, Kaohsiung Medical University, Kaohsiung, Taiwan; 2 Department of Ophthalmology, Kaohsiung Medical University Hospital, Kaohsiung Medical University, Kaohsiung, Taiwan; 3 Division of Hematology and Medical Oncology, Department of Internal Medicine, Pingtung Veterans General Hospital, Pingtung, Taiwan; 4 Department of Nursing, Meiho University, Pingtung, Taiwan; 5 School of Medicine, National Sun Yat-sen University, Kaohsiung, Taiwan; 6 School of Medicine, College of Medicine, Kaohsiung Medical University, Kaohsiung, Taiwan; 7 Tropical Medicine Center, Kaohsiung Medical University Hospital, Kaohsiung, Taiwan; 8 Division of Infectious Diseases, Department of Internal Medicine, Kaohsiung Medical University Hospital, Kaohsiung, Taiwan

**Keywords:** bendamustine, case report, cytomegalovirus retinitis, immunosuppression, mantle cell lymphoma, rituximab

## Abstract

**Background:**

Cytomegalovirus (CMV) retinitis following bendamustine-containing therapy is a rare opportunistic infection with limited published evidence, and its optimal management in human immunodeficiency virus (HIV)-negative patients remains poorly defined.

**Case presentation:**

A 29-year-old man with stage IV splenic mantle cell lymphoma (MCL), previously treated with R-CHOP followed by bendamustine–rituximab (BR) plus ibrutinib, developed progressive bilateral visual symptoms and abdominal pain approximately three months after completing BR therapy. Colonoscopic biopsy confirmed CMV colitis, and CMV retinitis was confirmed in the right eye, with subsequent virological confirmation of intraocular CMV involvement in the left eye. Despite initial induction therapy with intravenous ganciclovir followed by oral valganciclovir, right-eye CMV retinitis progressed, necessitating repeated intravitreal ganciclovir injections and re-induction with intravenous ganciclovir. Immunologic evaluation revealed profound cellular and humoral immune dysfunction, including severe CD4+ lymphopenia (109 cells/μL) and hypogammaglobulinemia, while HIV infection and lymphoma relapse were excluded. Intravenous immunoglobulin (IVIG) replacement therapy was initiated. Because of the prolonged ocular disease course and delayed immune reconstitution, extended oral valganciclovir maintenance and multiple intravitreal ganciclovir injections were required. Serial follow-up demonstrated gradual CD4+ T-cell recovery (109 to 352 cells/μL) and increasing circulating CD19+ B-cell proportions (4.9% to 13.7%), in parallel with sustained virological control. By June 2026, visual acuity remained preserved with no evidence of active retinitis, and both aqueous humor and blood CMV polymerase chain reaction (PCR) assays were undetectable. Given sustained retinal inactivity, virological suppression, and continued lymphoma remission, antiviral therapy was discontinued.

**Conclusions:**

This case highlights that cumulative therapy-associated immune dysfunction may predispose HIV-negative patients to CMV retinitis, with BR representing an important contributing exposure. For CMV retinitis in HIV-negative patients, treatment duration should be individualized according to ophthalmic findings, virological response, and stability of the underlying hematologic disease rather than relying solely on CD4+ T-cell counts.

## 1 Introduction

Mantle cell lymphoma (MCL) is a relatively uncommon subtype of B-cell non-Hodgkin lymphoma (NHL), with clinical behavior ranging from indolent disease to aggressive lymphoma with advanced nodal and extranodal involvement. Despite poor outcomes with conventional chemotherapy, survival has improved with the advent of treatment regimens specifically developed for MCL (1). Bendamustine is an alkylating agent with pharmacologic properties distinct from conventional alkylating agents (2). Bendamustine-based regimens are widely used for chronic lymphocytic leukemia (CLL) and B-cell lymphoid malignancies, including indolent B-cell NHL and MCL, because of their favorable efficacy and tolerability (3, 4). However, bendamustine is associated with profound and prolonged T-cell depletion, with grade 3–4 lymphopenia reported in 62–92% of patients (5). Lymphocyte and CD4+ T-cell recovery may require months to years after treatment completion and may be further delayed by concurrent or maintenance rituximab (6–11). Additionally, rituximab may contribute to prolonged immune dysfunction through sustained B-cell depletion and hypogammaglobulinemia (5).

Such treatment-associated immune dysfunction increases susceptibility to opportunistic infections in patients with lymphoid malignancies. In a large population-based study of older patients with indolent NHL, bendamustine exposure was associated with an approximately fourfold increased risk of cytomegalovirus (CMV) infection (12). CMV disease after bendamustine exposure may involve multiple organs, most commonly the lungs and gastrointestinal tract, while ocular involvement in the form of CMV retinitis remains an uncommon but recognized end-organ manifestation (13–15).

CMV retinitis following bendamustine-containing therapy remains rare, with limited published data on its clinical characteristics, treatment, and outcomes (16–27). In particular, the optimal duration of maintenance antiviral therapy and criteria for treatment discontinuation in human immunodeficiency virus (HIV)-negative patients remain poorly defined. Here, we report a case of CMV retinitis in a young man with splenic MCL following sequential lymphoma-directed therapies, including bendamustine–rituximab (BR), and discuss treatment-associated immune dysfunction and antiviral management of CMV retinitis in HIV-negative patients.

## 2 Case description

A 29-year-old man with glucose-6-phosphate dehydrogenase deficiency was diagnosed with stage IV splenic mantle cell lymphoma with bone marrow involvement. He had no history of ocular disease, surgery, or trauma. He initially received eight cycles of rituximab, cyclophosphamide, doxorubicin, vincristine, and prednisone (R-CHOP) from August 2023 to March 2024, achieving a partial response. Because of residual disease, he subsequently received six cycles of BR plus ibrutinib from May to December 2024. His clinical course is summarized in **Figure 1**.

**Figure 1:**
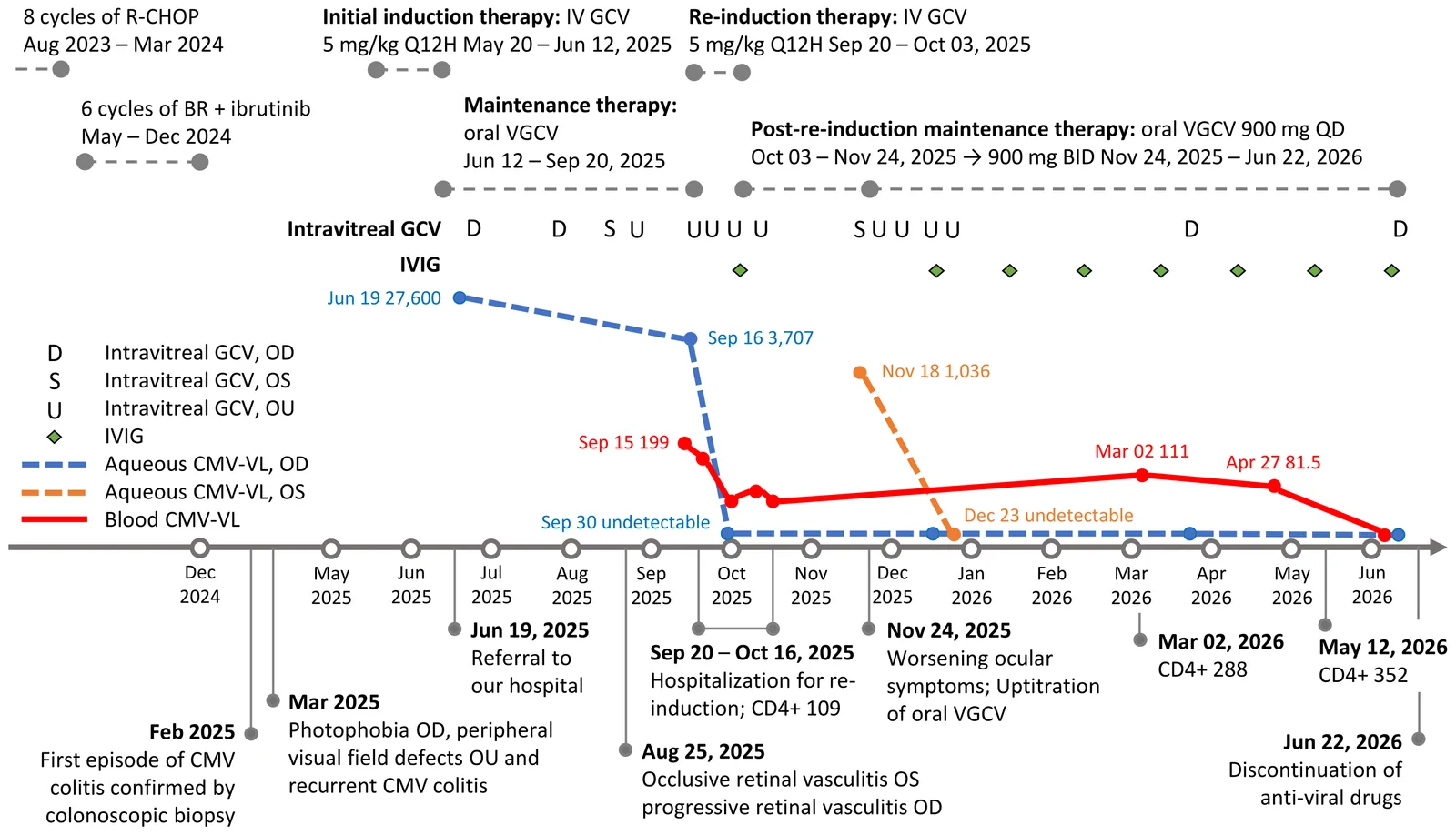
Timeline of key clinical events, treatment, and laboratory findings throughout the patient’s clinical course. Initial induction therapy of intravenous ganciclovir (GCV) 5 mg/kg every 12 hours for 24 days was followed by oral valganciclovir (VGCV) 450 mg twice daily. After referral to our hospital, oral VGCV was continued at 900 mg daily. Re-induction consisted of intravenous GCV 5 mg/kg every 12 hours for 14 days, followed by oral VGCV 900 mg daily, which was subsequently increased to 900 mg twice daily during worsening ocular disease. Intravitreal GCV was administered at 2 mg/0.04 mL per injection. Key aqueous humor CMV viral loads are shown for each eye. CMV-VL, cytomegalovirus viral load; IVIG, intravenous immunoglobulin.

Approximately two months after completing BR therapy, the patient developed his first episode of CMV colitis in February 2025 at Pingtung Veterans General Hospital (PVGH). In March 2025, he developed photophobia in the right eye. Over the following two months, he experienced progressive peripheral visual field defects, initially in the right eye and later in the left, accompanied by impaired color perception and right-eye floaters. In May 2025, recurrent abdominal pain and worsening visual symptoms prompted hospitalization at PVGH, where recurrent CMV colitis was confirmed by colonoscopic biopsy and CMV retinitis was clinically diagnosed. He received induction therapy with intravenous ganciclovir (5 mg/kg every 12 hours) for 24 days, followed by oral valganciclovir maintenance therapy (450 mg twice daily).

Despite antiviral therapy, the patient experienced progressive visual field deterioration and was referred to the ophthalmology department of Kaohsiung Medical University Hospital in June 2025. Best-corrected visual acuity was 20/20 bilaterally, with normal intraocular pressure and quiet anterior chambers without inflammatory cells. Aqueous humor CMV polymerase chain reaction (PCR) from the right eye showed a viral load of 27,600 IU/mL, while herpes simplex virus (HSV) PCR was undetectable. Color fundus photography demonstrated CMV retinitis involving the superior vascular arcade of the right eye; the active lesion was located outside Zone 1 and did not extend to the optic disc, without prominent retinal hemorrhage. No definite retinal necrosis or hemorrhage was observed in the left eye. Fluorescein angiography revealed associated retinal vasculitis predominantly in the right eye, with milder vascular changes in the left eye (**Figure 2**). Oral valganciclovir 900 mg daily was continued, and repeated intravitreal ganciclovir injections (2 mg/0.04 mL) were administered according to disease activity. By August 2025, however, fluorescein angiography demonstrated superonasal occlusive retinal vasculitis in the left eye, raising suspicion for CMV involvement, followed by vitreous opacity and progressive retinal vasculitis in the right eye (**Figures 3A, B**). No retinal break or detachment was identified in either eye. Because of progressive CMV retinitis in the right eye despite combined systemic and intravitreal antiviral therapy, together with persistent aqueous humor CMV PCR positivity (3,707 IU/mL) in September, he was admitted for re-induction treatment with intravenous ganciclovir.

**Figure 2:**
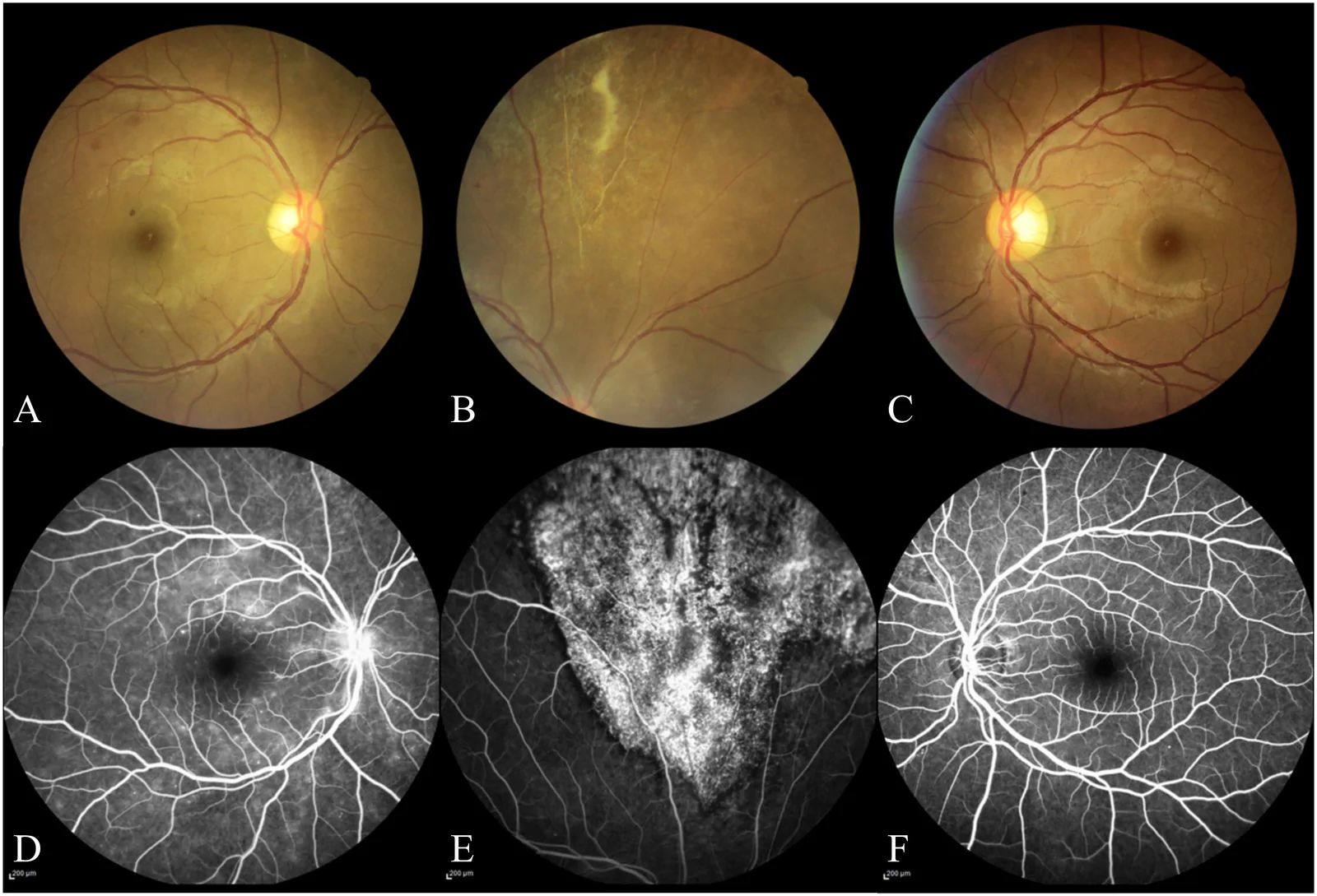
Multimodal retinal imaging at presentation. **(A, B)** Color fundus photographs of the right eye demonstrate retinal necrosis involving the superior vascular arcade, consistent with CMV retinitis, without prominent retinal hemorrhage. **(C)** Color fundus photograph of the left eye demonstrates no obvious retinal necrosis or hemorrhage. **(D–F)** Fluorescein angiography demonstrates retinal vasculitis of both eyes, more extensive in the right eye, corresponding to the area of retinal necrosis. No obvious retinal hemorrhage is observed in either eye.

**Figure 3:**
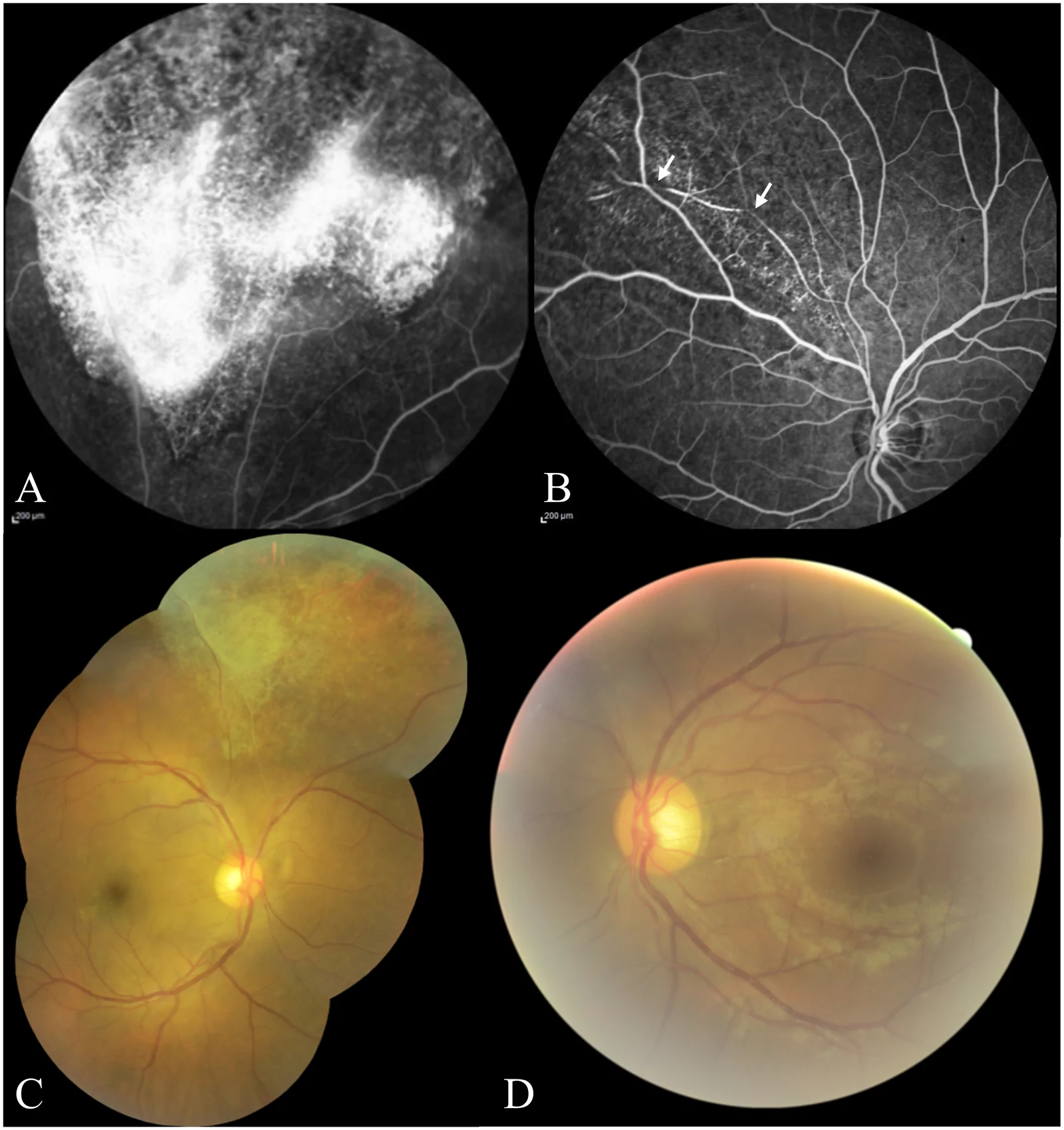
Follow-up retinal imaging demonstrating disease progression and clinical resolution. **(A, B)** Fluorescein angiography performed during follow-up (August 2025) demonstrates persistent CMV retinitis in the right eye and superonasal occlusive retinal vasculitis in the left eye (arrows). **(C, D)** Color fundus photographs in June 2026 show a healed CMV retinitis scar in the right eye and stable retinal findings in the left eye without evidence of active retinitis.

On admission, Humphrey 30–2 visual field testing demonstrated bilateral inferior visual field defects, more pronounced in the right eye. Intravenous ganciclovir (5 mg/kg every 12 hours) was administered from September 20 to October 3, 2025, together with weekly intravitreal ganciclovir injections. After 2 weeks, blood CMV DNA decreased from 129 to 40.1 IU/mL, and right-eye aqueous CMV PCR became undetectable. Following this clinical and virological response, he was transitioned to maintenance oral valganciclovir (900 mg daily). During intravenous ganciclovir therapy, the platelet count declined from a baseline of 223 × 10^9^/L to 168 × 10^9^/L but recovered after treatment completion without requiring interruption. Renal function remained preserved throughout antiviral therapy, with no dose reduction required. Antiviral resistance testing, including UL97 and UL54 genotyping, was not performed. Comprehensive immunologic evaluation revealed profound cellular and humoral immune dysfunction, including a CD4+ T-cell count of 109 cells/μL (CD4/CD8 ratio, 0.085), IgG <320 mg/dL, IgA 14 mg/dL, and IgM 21 mg/dL. HIV testing was negative. Given the persistent severe immunodeficiency, lymphoma relapse was considered. However, while abdominal computed tomography (CT) revealed splenomegaly, positron emission tomography/computed tomography (PET/CT) and hematologic evaluation demonstrated no evidence of recurrent disease. Intravenous immunoglobulin (IVIG) replacement therapy was therefore initiated for severe secondary hypogammaglobulinemia in the setting of serious infection. He was discharged in stable condition on maintenance oral valganciclovir (900 mg daily).

After discharge, the patient continued oral valganciclovir with regular follow-up by the ophthalmology, infectious diseases, and hematology teams. No interruption of oral valganciclovir therapy or adherence concerns were documented. During worsening ocular disease in November 2025, aqueous humor CMV PCR from the left eye was positive at 1,036.2 IU/mL, providing virological confirmation of intraocular CMV reactivation in the left eye. In response, additional intravitreal ganciclovir injections were administered, and oral valganciclovir was escalated from 900 mg once daily to 900 mg twice daily. Aqueous humor CMV PCR of the right eye remained undetectable after re-induction therapy, while the left-eye aqueous CMV viral load subsequently became undetectable by December 23, 2025. Monthly IVIG replacement was continued through June 2026 for persistent hypogammaglobulinemia. During subsequent follow-up, mild blurred vision with low-level CMV DNAemia (111 and 81.5 IU/mL in March and April 2026, respectively) prompted aqueous humor sampling and concurrent empiric intravitreal ganciclovir injections in March and June; right-eye aqueous CMV PCR remained undetectable on both occasions, with no objective evidence of recurrent active CMV retinitis. Serial immunologic evaluation showed CD4+ T-cell recovery from 109 cells/μL (CD4/CD8 ratio, 0.085) during hospitalization to 199 (0.11), 288 (0.21), and 352 cells/μL (0.24) at 1, 5, and 7 months after discharge, respectively. The proportion of circulating CD19+ B cells increased from 4.9% and 4.8% in September and November 2025 to 13.9% and 13.7% in March and June 2026, consistent with numerical B-cell reconstitution. Serum IgG levels increased from <320 mg/dL in September 2025 to 696 mg/dL in March and 676 mg/dL in June 2026 during ongoing IVIG replacement therapy, while IgA remained low; therefore, these IgG measurements could not be interpreted as evidence of endogenous humoral immune recovery. No evidence of lymphoma relapse was identified throughout follow-up.

By June 2026, visual field testing showed improvement. Visual acuity remained preserved, intraocular pressures were normal, and the anterior chambers remained quiet, with no evidence of active retinitis (**Figures 3C, D**). Both aqueous humor and blood CMV PCR assays were undetectable. Given sustained retinal inactivity, virological suppression, and continued remission of the underlying lymphoma, oral valganciclovir was discontinued on June 22, 2026. The patient remained clinically stable without recurrent CMV retinitis or lymphoma relapse during approximately two months of follow-up after antiviral discontinuation.

## 3 Discussion

We report a young patient with splenic MCL who developed recurrent CMV disease, including colitis and retinitis, following sequential lymphoma-directed therapies. Profound cellular and humoral immune dysfunction was considered multifactorial, with BR representing an important contributing exposure.

### 3.1 Prolonged immune dysfunction following bendamustine-based therapy

Bendamustine-based therapy can induce prolonged immune dysfunction characterized predominantly by persistent CD4+ T-cell depletion and, when combined with rituximab, additional humoral impairment. Gafter-Gvili et al. reported frequent grade 3–4 lymphopenia, greater lymphocyte depletion than with R-CHOP, and delayed immune reconstitution lasting months after treatment completion. Hypogammaglobulinemia has also been observed, although its pathogenesis is likely multifactorial, reflecting bendamustine-associated T-cell depletion, rituximab-associated B-cell depletion, and the underlying lymphoproliferative disorder (5). Previous studies have further demonstrated that absolute lymphocyte counts typically recover within approximately 7–9 months after bendamustine-based therapy (6), whereas CD4+ T-cell recovery may require 24 months or longer and may be further delayed by rituximab (6–8, 11). Importantly, absolute lymphocyte recovery does not reliably indicate cellular immune restoration, as approximately one-quarter of patients remain CD4+ lymphopenic despite normalized lymphocyte counts. CD4+ T-cell monitoring may therefore better reflect immune reconstitution (7, 8).

In our patient, CMV disease first manifested as colitis approximately two months after completion of BR therapy, followed by recurrent colitis and CMV retinitis over the subsequent months. Immunologic assessment during hospitalization for progressive ocular disease demonstrated profound CD4+ lymphopenia (109 cells/μL) and severe hypogammaglobulinemia, while HIV infection and lymphoma relapse were excluded, supporting treatment-associated delayed immune reconstitution as an important predisposing factor. Subsequent CD4+ T-cell recovery and increasing CD19+ B-cell proportions paralleled sustained virological remission, although serum IgG levels during IVIG replacement could not establish endogenous humoral recovery.

Other lymphoma-directed therapies may also have contributed. Rituximab induces profound and prolonged B-cell depletion and may result in hypogammaglobulinemia, potentially impairing antiviral immunity and delaying immune reconstitution (5, 28). Although CMV control primarily depends on T-cell immunity, persistent B-cell depletion has been proposed to indirectly impair T-helper-cell responses and viral antigen presentation (28). In a case series of non-transplant hematologic malignancies, 7 of 8 lymphoma patients who developed CMV infection had received rituximab-containing chemotherapy, although concomitant corticosteroid exposure precluded attribution to rituximab alone (29). Severe CMV end-organ disease has also been reported after rituximab maintenance (28), and CMV retinitis occurred in 5 of 138 patients receiving rituximab plus fludarabine-containing therapy, compared with none of 141 patients receiving comparable regimens without rituximab (30). Ibrutinib may also alter host immunity, although its effects differ from the profound and prolonged T-cell depletion associated with bendamustine. Studies in CLL have shown normalization of dysregulated T-cell subsets, restoration of T-cell function, and partial humoral reconstitution during prolonged therapy (31–33). Nevertheless, opportunistic infections, particularly invasive fungal infections, have been reported (34, 35). CMV disease has rarely been described, including CMV proctitis (36), whereas, to our knowledge, CMV retinitis specifically associated with ibrutinib has not been reported. Conventional cytotoxic chemotherapy may also have contributed to cumulative immunosuppression. Although CMV infection has been reported in patients receiving CHOP or CHOP-like regimens, evidence supporting CHOP itself as a specific risk factor for CMV disease remains limited (29, 37). Nevertheless, corticosteroids may contribute to CMV susceptibility through suppression of cell-mediated immunity, including preferential CD4+ T-cell depletion and a reduced CD4/CD8 ratio (38). In a retrospective cohort of non-transplant lymphoma patients, steroid pretreatment and exposure to more than two therapeutic regimens were independently associated with increased risk (37). Thus, prior R-CHOP therapy, particularly its corticosteroid component, may have contributed to immune dysfunction in our patient. The profound immunosuppression was therefore considered multifactorial, reflecting cumulative effects of sequential lymphoma-directed therapies. Nevertheless, the temporal association with BR, its established effects on CD4+ T-cell recovery, and previous reports of CMV retinitis following bendamustine-containing regimens support BR as an important contributing exposure.

### 3.2 Clinical characteristics of CMV retinitis following bendamustine-containing therapy

Although CMV retinitis most commonly occurs in patients with advanced HIV infection, CMV retinitis following bendamustine-containing therapy remains rare. A literature search of PubMed/MEDLINE, Embase, Scopus, and Google Scholar through September 30, 2026, using combinations of *bendamustine*, *cytomegalovirus/CMV*, and *retinitis/ocular/intraocular*, supplemented by citation tracking, identified 23 previously published patient-level cases across 12 studies (16–27) (**Table 1**).

**Table 1:** Published patient-level cases of CMV retinitis following bendamustine-containing therapy.

Study	Patient	Disease/prior therapy	B regimen (cycles)	CMV disease	Onset	Treatment	Outcome
Hosoda 2013 (16)	51M	FL R-CHOP, R-FND	B (3)	Retinitis (laterality NR), hepatitis, enteritis	10 weeks after B initiation	GCV, FOS, VGCV	Died after bone marrow transplant
Shah 2015 (17)	67F	MCL NR	BR (NR)	Bilateral retinitis, colitis	NR	IV + intravitreal FOS	Retinitis arrested; poor visual outcome
Yamasaki 2017 (18)	Age: 71, 81, 88 1 M, 2 F	FL (n =1), tFL (n =2) Regimens NR; prednisolone in 2/3	B (4-6) R in 1/3	Retinitis (laterality NR)	5, 10, 36 months after B initiation	IV + intravitreal GCV	Retinitis improved
Cona 2019 (19)	75M	LPL/WM NR	BR (6)	Bilateral retinitis, encephalitis, gastritis, colitis	≈3 months after completion of BR	IV GCV → FOS induction; VGCV maintenance/re-induction	Partial visual recovery
Imai 2021 (20)	68M	FL no prior therapy	BR (6)	Bilateral retinitis	9 months after B initiation	IV + intravitreal GCV; VGCV maintenance	Recurrent retinitis; loss of light perception OU
	83M	MALT lymphoma dexamethasone, R, cyclophosphamide	BR (6)	Left retinitis	15 months after B initiation	IV + intravitreal GCV; VGCV maintenance	No recurrence; loss of light perception OS
Wu 2022 (21)	49F	FL-DLBCL R-CHOP, R-FND, Hyper-CVAD, R-DHAP, DIME, R-GEMOX	BR (1)	Bilateral retinitis	NR	Systemic + intravitreal antiviral (agent NR)	Poor vision persisted
	69F	Myeloma VTD, VRD, VCMD	B (2)	Bilateral retinitis	NR	Systemic antiviral (agent NR)	Good visual improvement
	58F	AITL CHOP, FND, SIMPLE	B (4)	Left retinitis	NR	Systemic + intravitreal antiviral (agent NR)	Good visual improvement
	49F	MCL NR	O-BAC (3)	Bilateral retinitis, duodenitis, colitis	NR	Systemic antiviral (agent NR)	Some visual improvement
Tay 2022 (22)	72M	B-cell LPD no prior therapy	GB (NR)	Retinitis (laterality NR)	NR	NR	Alive
Saburi 2023 (23)	65F	FL no prior therapy	BR (4)	Retinitis (laterality NR)	234 days after B initiation	NR	CMV infection recovered
	54M	FL no prior therapy	BR (6)	Retinitis (laterality NR)	352 days after B initiation	NR	CMV infection recovered
	69M	FL no prior therapy	GB (6)	Retinitis (laterality NR)	280 days after B initiation	NR	CMV infection not recovered
	58F	FL no prior therapy	GB (4)	Retinitis (laterality NR), gastritis	116 days after B initiation	NR	CMV infection recovered
Alkhuraiji 2023 (24)	58F	DLBCL NR	Pola-BR (5)	Bilateral retinitis	5 months after treatment initiation	GCV → VGCV; intravitreal GCV/FOS	Stable retinitis; VA remained hand motion OU
	76M	MZL NR	BR (4)	Bilateral retinitis	4 months after treatment initiation	VGCV/FOS; intravitreal GCV	Visual deterioration; vitreous hemorrhage
Passarin 2024 (25)	75F	FL NR	BR (NR)	Bilateral retinitis	NR	VGCV; intravitreal FOS	No recurrence; severe visual loss OD
	54F	FL NR	BR (NR)	Retinitis (laterality NR), GI and neurological CMV disease	NR	IV GCV → VGCV	Recurrence; severe visual loss
Jobanputra 2025 (26)	67M	Low-grade B-cell NHL NR	BR (6)	Bilateral retinitis	~4 months after BR completion	Systemic + intravitreal GCV	No CMV reactivation; vision improved
Clavell 2026 (27)	60F	FL no prior therapy	BR (NR)	Bilateral retinitis	>1 year after completion of B	IV GCV; oral VGCV; intravitreal GCV + FOS	Quiescent OU; VA 20/400 OD, 20/20 OS
This study	29M	MCL R-CHOP	BR+ibrutinib (6)	Right retinitis, left-eye CMV reactivation, colitis	≈3 months after completion of BR	IV GCV induction/re-induction, oral VGCV maintenance, intravitreal GCV	Preserved VA; no evidence of active retinitis

AITL, angioimmunoblastic T-cell lymphoma; B, bendamustine; BR, bendamustine-rituximab; CMV, cytomegalovirus; DIME, dexamethasone, ifosfamide, methotrexate and etoposide; DLBCL, diffuse large B-cell lymphoma; F, female; FL, follicular lymphoma; FOS, foscarnet; GB, obinutuzumab-bendamustine; GCV, ganciclovir; GI, gastrointestinal; Hyper-CVAD, cyclophosphamide, vincristine, doxorubicin, dexamethasone, methotrexate, cytarabine and methylprednisolone; IV, intravenous; LPD, lymphoproliferative disease; LPL/WM, lymphoplasmacytic lymphoma/Waldenstrom macroglobulinemia; M, male; MALT, mucosa-associated lymphoid tissue; MCL, mantle cell lymphoma; MZL, marginal zone lymphoma; NHL, non-Hodgkin lymphoma; NR, not reported; O-BAC, obinutuzumab-bendamustine-cytarabine; OD, right eye; OS, left eye; OU, both eyes; Pola-BR, polatuzumab vedotin, bendamustine and rituximab; R-CHOP, rituximab, cyclophosphamide, doxorubicin, vincristine and prednisolone; R-DHAP, rituximab, dexamethasone, cytarabine and cisplatin; R-FND, rituximab, fludarabine, mitoxantrone and dexamethasone; R-GEMOX, gemcitabine, methotrexate and oxaliplatin; SIMPLE, dexamethasone, ifosfamide, methotrexate, cisplatin, L-asparaginase and etoposide; tFL, transformed follicular lymphoma; VA, visual acuity; VCMD, bortezomib, cyclophosphamide, melphalan and dexamethasone; VGCV, valganciclovir; VRD, bortezomib, lenalidomide and dexamethasone; VTD, bortezomib, thalidomide and dexamethasone.

Most patients were older adults with hematologic malignancies, most commonly follicular lymphoma; two patients with MCL have been previously reported (17, 21). Treatment exposure was heterogeneous, with several cases occurring after first-line bendamustine-based therapy (20, 22, 23). Reported onset ranged from approximately 10 weeks to 36 months after bendamustine initiation, with some cases developing more than one year after treatment completion. Bilateral ocular involvement predominated among cases with reported laterality. Systemic antiviral therapy, often combined with intravitreal treatment, was used in most cases. Detailed dosing and treatment duration were inconsistently reported. Visual outcomes ranged from improvement or disease control to severe persistent visual loss.

Compared with previously reported cases, our patient was substantially younger and adds a detailed case of CMV retinitis in splenic MCL following BR-containing therapy. Our report provides longitudinal virological and immunological monitoring over more than one year, demonstrating gradual CD4+ T-cell recovery and increasing circulating CD19+ B-cell proportions in parallel with sustained CMV control. Prolonged systemic and intravitreal therapy preserved visual acuity and ultimately allowed valganciclovir discontinuation.

### 3.3 Antiviral management and treatment discontinuation of CMV retinitis in HIV-negative patients

The optimal duration of maintenance therapy for CMV retinitis in HIV-negative patients remains poorly defined. Current HIV guidelines recommend stopping maintenance therapy after at least 3 months of anti-CMV treatment, with inactive retinitis and a CD4+ T-cell count ≥100 cells/μL for at least 3 months on antiretroviral therapy (39). These criteria cannot be directly extrapolated to HIV-negative patients. Tasiopoulou et al. proposed continuing antiviral therapy in lymphoma-associated CMV retinitis until ocular lesions become inactive, CMV viremia has resolved, and the hematologic disease remains stable (40). These principles are consistent with the recent Asia-Pacific consensus, emphasizing ophthalmic inactivity and control of the predisposing systemic condition. Although intraocular CMV PCR is not recommended as the primary criterion for treatment discontinuation, it may provide valuable adjunctive information for monitoring virological response (41). For persistent or recurrent disease, delayed immune reconstitution is an important consideration, particularly when CD4+ T-cell counts remain below approximately 50–100 cells/μL. In this setting, prolongation of anti-CMV therapy may be preferable to an immediate switch in antiviral agents, provided that adherence, inadequate intraocular drug exposure, and antiviral resistance have been assessed (41).

Our management strategy closely paralleled these recommendations. Because of the prolonged ocular disease course and delayed immune reconstitution, extended oral valganciclovir maintenance and repeated intravitreal ganciclovir injections were required. Antiviral therapy was discontinued only after retinal lesions had remained inactive, both aqueous humor and blood CMV PCR became undetectable, and serial hematologic evaluation confirmed sustained remission of the underlying lymphoma.

### 3.4 Differential diagnosis and ocular inflammation during immune recovery

Differential diagnoses included HSV- or varicella-zoster virus (VZV)-associated acute retinal necrosis (ARN), toxoplasma retinochoroiditis, and intraocular lymphoma. HSV PCR of right-eye aqueous humor was undetectable. Although VZV PCR was not performed, the absence of anterior chamber inflammation or rapidly progressive circumferential peripheral retinal necrosis was not typical of classic ARN. Toxoplasma-specific testing was unavailable, but the fundus lacked typical focal necrotizing retinochoroiditis adjacent to a pre-existing scar. Intraocular lymphoma was also considered given the underlying MCL; however, PET/CT and hematologic evaluation showed no evidence of lymphoma relapse. Although these findings could not definitively exclude intraocular lymphoma, the retinal findings together with strongly positive aqueous humor CMV PCR supported CMV retinitis as the primary diagnosis.

Immune recovery uveitis (IRU) may manifest with vitritis, retinal vasculitis, or other inflammatory findings during immune reconstitution after CMV retinitis and can be difficult to distinguish from active infection (41). In our patient, intraocular CMV reactivation in November 2025 was supported by renewed aqueous humor CMV PCR positivity. In contrast, subsequent mild ocular symptoms in March and June 2026 occurred during progressive CD4+ T-cell recovery without documented new retinal necrosis, extension of an active retinitis border, or renewed CMV PCR positivity. Therefore, IRU or other post-infectious inflammatory sequelae, including persistent retinal vasculitis, were considered alternative explanations for these later episodes, although available clinical data did not permit definitive distinction.

### 3.5 Limitations

Several limitations should be acknowledged. Pretreatment CD4+ T-cell counts and immunoglobulin levels were unavailable, precluding exclusion of pre-existing cellular or humoral immune dysfunction. Serum IgG levels were confounded by IVIG replacement and could not establish endogenous humoral recovery. CMV-specific cellular immune assays were unavailable, limiting assessment of CMV-specific immune reconstitution. Antiviral resistance testing, including UL97 and UL54 genotyping, was also not performed; therefore, the contribution of antiviral resistance to the persistent or progressive ocular disease could not be determined. Finally, the approximately two-month follow-up after valganciclovir discontinuation limits conclusions regarding longer-term outcomes.

## 4 Take-away lessons

CMV retinitis is a rare but vision-threatening opportunistic infection that may occur following bendamustine-based therapy. Cumulative cellular and humoral immune dysfunction from sequential lymphoma-directed therapies should be considered, with BR representing an important contributing exposure. In HIV-negative patients, antiviral management should be individualized according to clinical disease activity and virological response rather than CD4+ T-cell counts alone.

## 5 Patient perspective

*The following patient perspective was translated into English by the authors from the patient’s own statement.*

When my vision began to worsen in both eyes, I felt frightened and uncertain about the future. I have a two-year-old daughter, and the thought that I might not be able to clearly see her face or watch her grow up was especially difficult for me. The prolonged treatment also brought emotional and financial stress, although private insurance helped reduce part of the financial burden.

With the coordinated care of the ophthalmology, infectious diseases, and hematology teams, my condition gradually stabilized. I was relieved to learn that my lymphoma had not relapsed and that the infection was related to the weakened immunity following my previous cancer treatment. As my vision improved, I regained confidence about the future. More than anything, I hope to remain healthy, preserve my vision, and watch my daughter grow up.

## Data Availability

The original contributions presented in the study are included in the article/**Supplementary Material**. Further inquiries can be directed to the corresponding author.
