# Supplementary material for: Case Report: Cytomegalovirus retinitis in an HIV-negative patient with splenic mantle cell lymphoma treated with bendamustine-rituximab
Source: Front Immunol. 2026 Oct 28;17:1979881. doi: 10.3389/fimmu.2026.1979881 (PMC13751967; doi:10.3389/fimmu.2026.1979881)
Supplement: Supplementary file 1 [file Supplementaryfile1.pdf]

Supplementary Material

Supplementary Table 1. Antiviral treatment course and clinical rationale for treatment modification

Antiviral regimen	Dose	Frequency	Route	Duration	Clinical rationale
Induction with ganciclovir	5 mg/kg	Every 12 hour	Intravenous dripping	May 20 – Jun 12, 2025	Induction treatment for CMV end-organ disease
maintenance with valganciclovir	450 mg	Twice daily	Oral	Jun 12 – Jun 17, 2025	Transition to oral maintenance after initial induction
maintenance with valganciclovir	900 mg	Once daily	Oral	after referral to our hospital Jun 17 – Sep 20, 2025	Continued maintenance after referral to our hospital
Re-induction with ganciclovir	5 mg/kg	Every 12 hour	Intravenous dripping	Sep 20 – Oct 03, 2025	Progressive right-eye CMV retinitis with persistent aqueous CMV PCR positivity despite systemic and intravitreal therapy
Post-re-induction maintenance with valganciclovir	900 mg	Once daily	Oral	Oct 03 – Nov 24, 2025	Transition to maintenance after clinical/virological response to re-induction and undetectable OD aqueous CMV PCR
	900 mg	Twice daily	Oral	Nov 24, 2025 – Jun 22, 2026	Escalation for intraocular CMV reactivation with OS aqueous CMV PCR positivity
Intravitreal ganciclovir	2 mg/0.04 mL	Single injection	Intravitreal	Number of injections to right eye solely: 4<br>left eye solely: 2<br>both eyes: 9	Administered according to ocular disease activity; later injections prompted by recurrent/worsening ocular findings or symptoms

Supplementary Table 2. Longitudinal key laboratory parameters during antiviral treatment and follow-up

**Date**	**Laboratory parameter (institutional reference range)**							
	WBC	Hb	PLT	NEUT	AST	ALT	BUN	Cr
	(3,920–9,240 /μL)	(13.1–17.9 g/dL)	(171,000–400,000 /μL)	(37.9-76.8%)	(11–34 IU/L)	(< 45 IU/L)	(8.4–25.7 mg/dL)	(0.60–1.20 mg/dL)
Sep 15, 2025	10690	14.9	223000	62.0	26	33	8.5	0.60
Sep 22, 2025	6510	14.5	208000	48.2	NT	NT	NT	NT
Sep 23, 2025	6410	14.2	202000	71.0	NT	NT	NT	NT
Sep 26, 2025	7480	14.3	181000	54.7	23	25	10.5	0.56
Sep 30, 2025	8360	14.7	175000	71.9	27	29	14.4	0.62
Oct 03, 2025	7820	14.6	168000	65.0	NT	NT	NT	NT
Oct 08, 2025	7460	14.6	166000	NT	NT	NT	NT	NT
Oct 15, 2025	4890	14.2	175000	NT	NT	NT	NT	NT
Nov 03, 2025	6670	14.5	179000	58.6	NT	NT	NT	NT
Dec 01, 2025	8140	15.1	184000	NT	NT	NT	NT	NT
Dec 15, 2025	7060	15.1	230000	56.5	NT	NT	NT	NT
Mar 02, 2026	6740	14.8	182000	60.4	NT	NT	NT	NT
Jun 04, 2026	6480	15.4	171000	59.0	NT	39	15.6	0.76

ALT, alanine aminotransferase; AST, aspartate aminotransferase; BUN, blood urea nitrogen; Cr, creatinine; Hb, hemoglobin; NEUT, neutrophil; NT, not tested; PLT, platelet; WBC, white blood cell.
